# Supplementary material for: Metatranscriptomic Analysis of Oropharyngeal Samples Reveals Common Respiratory Viruses and a Potential Interspecies Transmitted Picobirnavirus in the Wayuu Population, La Guajira, Colombia
Source: Viruses. 2025 Oct 21;17(10):1397. doi: 10.3390/v17101397 (PMC12568090; doi:10.3390/v17101397)
Supplement: Supplementary file 1 [file viruses-17-01397-s001.zip › Supplementary figure S1 and S2.pdf]

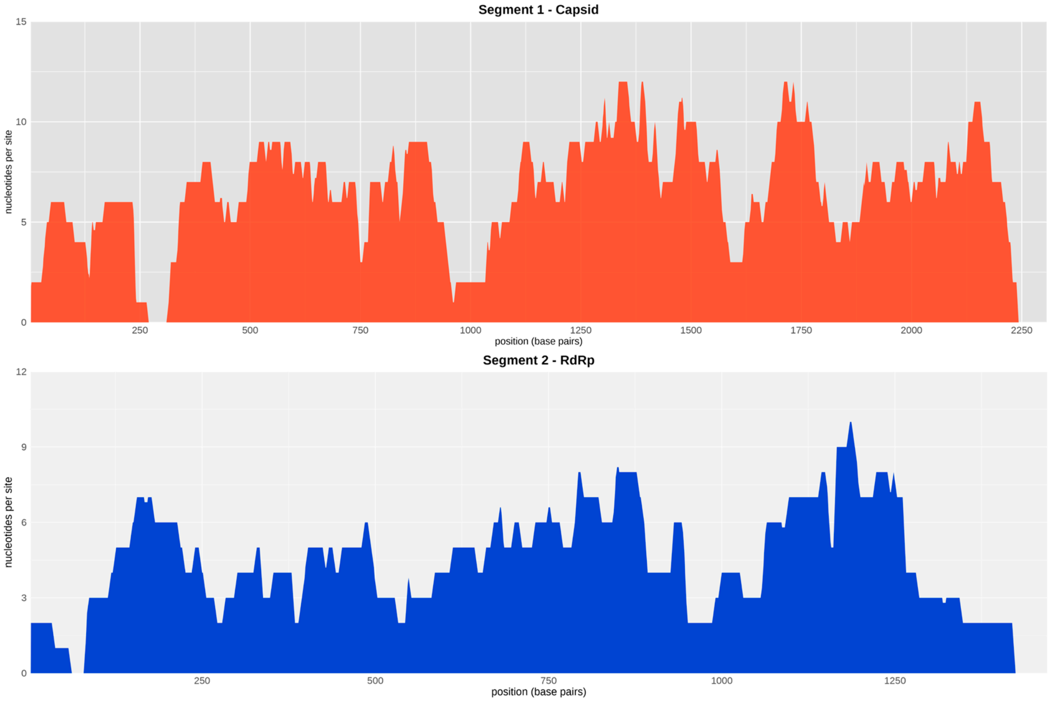

Figure S1. Coverage and sequencing depth plots obtained from mtNGS data for PBV segments: Segment 1 encoding the capsid protein (red) and Segment 2 encoding the RNA-dependent RNA polymerase (blue).

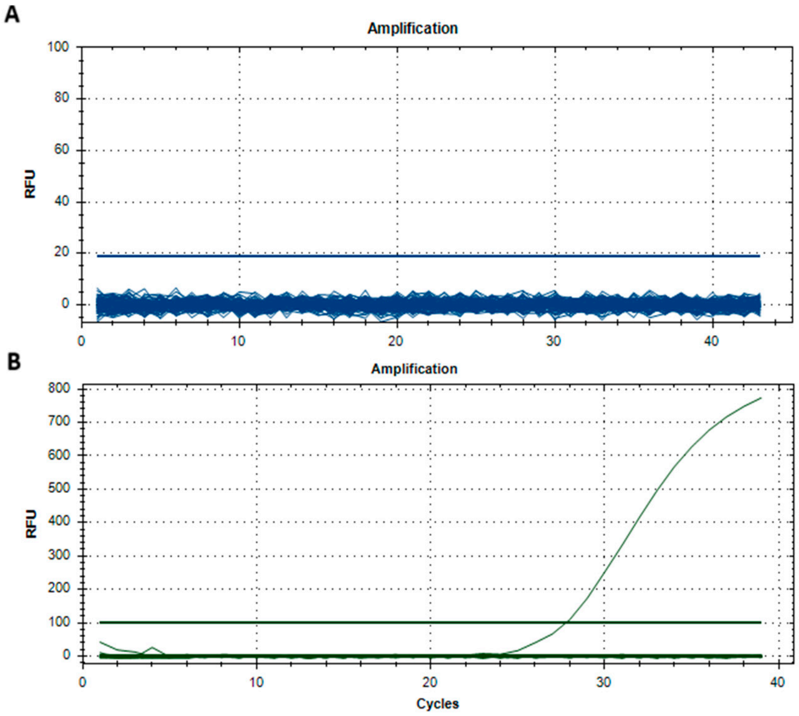

Figure S2: Real-time qPCR result for sample COL_INS-GME-06. (A) Detection of PBVcapsid gene using FAM-labeled primers previously described by Berg et al. [17]. (B) Amplification of the human RNase P gene with a HEX-labeled probe confirming RNA quality and sample integrity, serving as an internal control.
